# Lack of validated patient‐reported outcome tools persists in paediatric and adolescent hip arthroscopy—A systematic review

**DOI:** 10.1002/ksa.12603

**Published:** 2025-01-29

**Authors:** Ayomide Michael Ade‐Conde, Brendan Amoyaw, Yoan Bourgeault‐Gagnon, Hassaan Abdel Khalik, Nicole Simunovic, Olufemi R. Ayeni

**Affiliations:** ^1^ School of Medicine Royal College of Surgeons in Ireland Dublin Ireland; ^2^ Department of Health Science, Faculty of Health Science McMaster University Hamilton Ontario Canada; ^3^ Division of Orthopaedic Surgery, Department of Surgery McMaster University Hamilton Ontario Canada

**Keywords:** clinimetrics, femoroacetabular impingement, hip arthroscopy, patient‐reported outcome measures, paediatrics

## Abstract

**Purpose:**

This systematic review aimed to (1) identify commonly used patient‐reported outcome (PRO) tools in paediatric hip arthroscopy and (2) assess whether the PROs used in this population have been formally validated.

**Methods:**

Two systematic searches of MEDLINE, Embase and CENTRAL, from inception to 31 March 2024 and 22 August 2024, respectively, followed the Preferred Reporting Items for Systematic Reviews and Meta‐analyses guidelines. The first search identified PRO instruments used in studies on hip arthroscopy in patients aged 19 and under. The second focused on the clinimetric properties of these tools in paediatric hip arthroscopy. PRO utilization was stratified by pathology, trends over time and publication type. Use of the Consensus‐based Standards for the Selection of Health Measurement Instruments tool, and a descriptive analysis, were planned to assess the eligible clinimetric studies.

**Results:**

Fifty‐seven studies were included, identifying 10 hip‐specific and 5 nonspecific PROs. The second search did not identify any clinimetric studies on these tools used in paediatric patients. The most commonly reported hip‐specific PRO were the modified Hip Harris Score (*n* = 48), the Hip Outcome Score–Sport‐Specific Subscale (*n* = 25) and the Non‐Arthritic Hip Score (*n* = 20). Hip arthroscopy was used to treat over seven different conditions, with femoroacetabular impingement being the most common (*n* = 41, 77%). Between 2005 and 2024, the variety of hip‐specific PROs increased, with seven new ones introduced by 2019–2024. Additionally, this study found a relatively equal distribution of outcomes across presentation abstracts and manuscripts.

**Conclusions:**

The key finding of this study is the ongoing lack of hip‐specific PRO tools in the paediatric hip arthroscopy literature, with reliance on adult‐derived instruments. The absence of clinimetric studies and heterogeneity in PRO use emphasises the need for standardized, paediatric‐specific tools. Developing and validating such instruments should be prioritized to ensure accurate, age‐appropriate outcome assessment and care.

**Level of Evidence:**

Level III.

**Abbreviations**
ACL: anterior cruciate ligament
COSMIN: Consensus‐based Standards for the Selection of Health Measurement Instruments
FAIS: femoroacetabular impingement syndrome
HAGOS: Copenhagen Hip and Groin Outcome Score
HHS: Hip Harris Score
HOOS: Hip disability and Osteoarthritis Outcome Score
HOS‐ALD: Hip Outcome Score–Activities of Daily Living Subscale
HOS‐SS: Hip Outcome Score–Sport‐Specific Subscale
iHOT‐12: International Hip Outcome Tool‐12
iHOT‐33: International Hip Outcome Tool‐33
MINORS: Methodological Index for Non‐Randomized Studies
NAHS: Non‐Arthritic Hip Score
OSF: Open Science Framework
pedi‐IKDC: modified International Knee Documentation Committee Subjective Knee Form
PRISMA: Preferred Reporting Items for Systematic Reviews and Meta‐analysis
PRO: patient‐reported outcome
SCFE: slipped capital femoral epiphysis
SF‐12: 12‐Item Short Form
TAS: Tegner Activity Scale
UCLA Activity Score: University of California, Los Angeles Activity Score
VAS: visual analogue scale for Pain
WOMAC: Western Ontario and McMaster Universities Osteoarthritis Index

## INTRODUCTION

Hip arthroscopy has rapidly evolved over the past two decades from a primarily diagnostic tool to an effective intervention for treating various intra‐articular hip pathologies in children and adolescents [27]. This minimally invasive technique has enabled a better understanding of intra‐articular hip pathologies, preventing long‐term hip joint damage, and improving function in many paediatric surgical indications [28]. As a result, the incidence of hip arthroscopy procedures in adolescent patients has more than quintupled between 2008 and 2018 in the United States [13]. As the use of arthroscopy continues to rise within the paediatric population, it is important to evaluate the outcomes of this intervention. Patient‐reported outcomes (PROs) are considered the reference standard in orthopaedic post‐operative outcome evaluation, however, concerns have been raised regarding their validity in paediatric patients, particularly in younger children or those lacking the maturity to fully comprehend the questionnaires [2].

Several PRO instruments have been developed to guide the post‐surgical management and evaluation of hip pathologies. However, most of these disease‐ and joint‐specific tools, such as the Western Ontario and McMaster Universities Arthritis Index (WOMAC) and Hip Harris Score (HHS), were designed for osteoarthritic conditions which are significantly more prevalent in adult populations. While these hip‐specific PROs have undergone clinimetric analyses to validate their use in the arthroscopic treatment of various hip pathologies in adult patients, the validity of the aforementioned PROs remains unclear in the paediatric population [17, 26]. Using adult PRO instruments in paediatric patients without prior demographic‐specific validation can lead to inaccurate assessments and potentially misguided treatment decisions [32, 33]. A previous systematic review published a decade ago identified six hip‐specific PROs reported in the paediatric femoroacetabular impingement syndrome (FAIS) literature, yet none of these tools had been validated in a paediatric population [6]. The authors underscored the necessity of developing paediatric‐specific tools to more appropriately evaluate outcomes in this population. Furthermore, similar challenges have been documented in other orthopaedic patient populations such as adolescents with anterior cruciate ligament (ACL) injuries. A review identifying PROs used to evaluate paediatric patients after ACL injury found that no consensus existed in the evaluation of these injuries among children [34]. Despite the recognition of these concerns, the use of adult‐derived PRO scores for paediatric hip arthroscopy seems to persist, warranting further investigation.

Thus, this systematic review sought to identify gaps in the validation and appropriateness of current PRO tools for younger patients. Specifically, it aimed to evaluate the current state of outcome reporting in the paediatric hip arthroscopy literature and to identify any potential paediatric‐specific tools that may have been developed since prior reviews. Secondarily, it aimed to assess whether PROs used in the paediatric hip arthroscopy patient population have been formally validated. By addressing these questions, this study aimed to provide stakeholders with important information in regard to selecting the appropriate PRO tools to assess the use of hip arthroscopy among paediatric patients.

## METHODS

This review was performed according to the guidelines set out by the Cochrane Handbook and is reported according to the Preferred Reporting Items for Systematic Reviews and Meta‐analysis (PRISMA) [15, 23]. The protocol was registered on Open Science Framework (OSF) (Center for Open Science) before conducting the search [3].

### Comprehensive search strategy

We conducted two literature searches across three electronic databases—MEDLINE, Embase, and CENTRAL—from their inception to 31 March 2024 and 22 August 2024, for two subsequent and interdependent searches (A.M.A). The purpose of the first search was to identify the hip‐specific and non‐specific PRO tools utilized in studies assessing hip arthroscopy in paediatric patients. The search strategy was based on the key concepts ‘Hip’, ‘Arthroscopy’ and ‘Paediatric’ (Supporting Information S1: Table S1).

Using the PRO tools identified from the first literature search, a second search was performed to identify studies reporting the clinimetric characteristics of these tools, specifically in paediatric patients undergoing hip arthroscopy. The search strategy was based on the key concepts ‘Hip’, ‘Arthroscopy’, ‘clinimetric’ and ‘Paediatric’ (Supporting Information S1: Table S1).

The inclusion criteria for the first search in this review were: (1) hip arthroscopy for any indication, (2) paediatric and adolescent patient populations, aged 19 years old or less, (3) report a PRO, (4) levels of evidence I–IV including a minimum of five patients, and (5) studies published in English. Exclusion criteria were: (1) literature in the form of review articles, meta‐analyses, case reports, commentary and surveys and (2) studies assessing mixed groups of paediatric and adult patients.

The inclusion for the second search in this review was: (1) studies had to include at least one of the PROs identified from the first search, (2) assessment of the PRO specifically in a paediatric population, aged 19 years old or less, and (3) reporting of a clinimetric characteristic of the instrument(s).

### Study screening

Two reviewers (A.M.A and B.A), independently performed the title and abstract as well as the full‐text screening phases of the review. Conflicts at the title and abstract stage were automatically advanced to the full‐text stage to avoid premature exclusions of studies, while conflicts at the full‐text stage were resolved by a third reviewer (Y.B.G).

### Data abstraction from included studies

Two reviewers (A.M.A and B.A) independently abstracted the data from all of the included studies, while a third reviewer (Y.B.G) reviewed the accuracy of their data. The data was abstracted into a Covidence extraction template designed a priori. The following data was abstracted from the studies: study characteristics, number of patients, patient demographics, follow‐up length, as well as details of the PRO measures that were used. Included studies were classified into eight groups based on the reported hip pathology: (1) FAIS and/or labral tear, (2) Hip dysplasia or borderline dysplasia, (3) Hip septic arthritis, (4) Tuberculosis of the hip joint, (5) Legg‐Calve‐Perthes, (6) Slipped capital femoral epiphysis (SCFE), (7) Traumatic hip dislocation and (8) Multiple indications.

The methodological quality of the studies identified in the initial search was assessed independently by two reviewers using the Methodological Index for Non‐Randomized Studies (MINORS), with conflicts resolved by a third reviewer [29]. A critical risk of bias assessment was planned for eligible clinimetric studies using the Consensus‐based Standards for the Selection of Health Measurement Instruments (COSMIN) tool, which provides a standardized approach for evaluating the reliability and validity of health measurement instruments [24].

### Outcomes assessed

The primary outcome of this study was to identify the PRO scores that are most commonly used in paediatric patients following arthroscopic management of hip pathologies, their distribution across various pathologies, as well as to assess any temporal trends in their usage. The secondary outcome was to identify the validity of the hip‐specific outcome tools used in paediatric populations.

### Statistical analysis

Descriptive statistics, including pooled means and frequencies, were calculated for various study characteristics and PROs using a pairwise inclusion method. Relevant study characteristics and outcomes were also stratified by publication type, pathology and publication year. Temporal trends in the use of hip‐specific outcomes over 5‐year intervals from 2005 to 2024 were evaluated. Three subgroup analyses of the PROs reported were performed, categorized by publication types, pathology studied and 5‐year intervals. The clinimetric properties of the targeted PROs were planned to be assessed using descriptive statistics and qualitative analysis. All analyses were performed using Microsoft Excel for Mac v.16.92 (Microsoft Corporation).

## RESULTS

### Included studies

**Figure 1:**
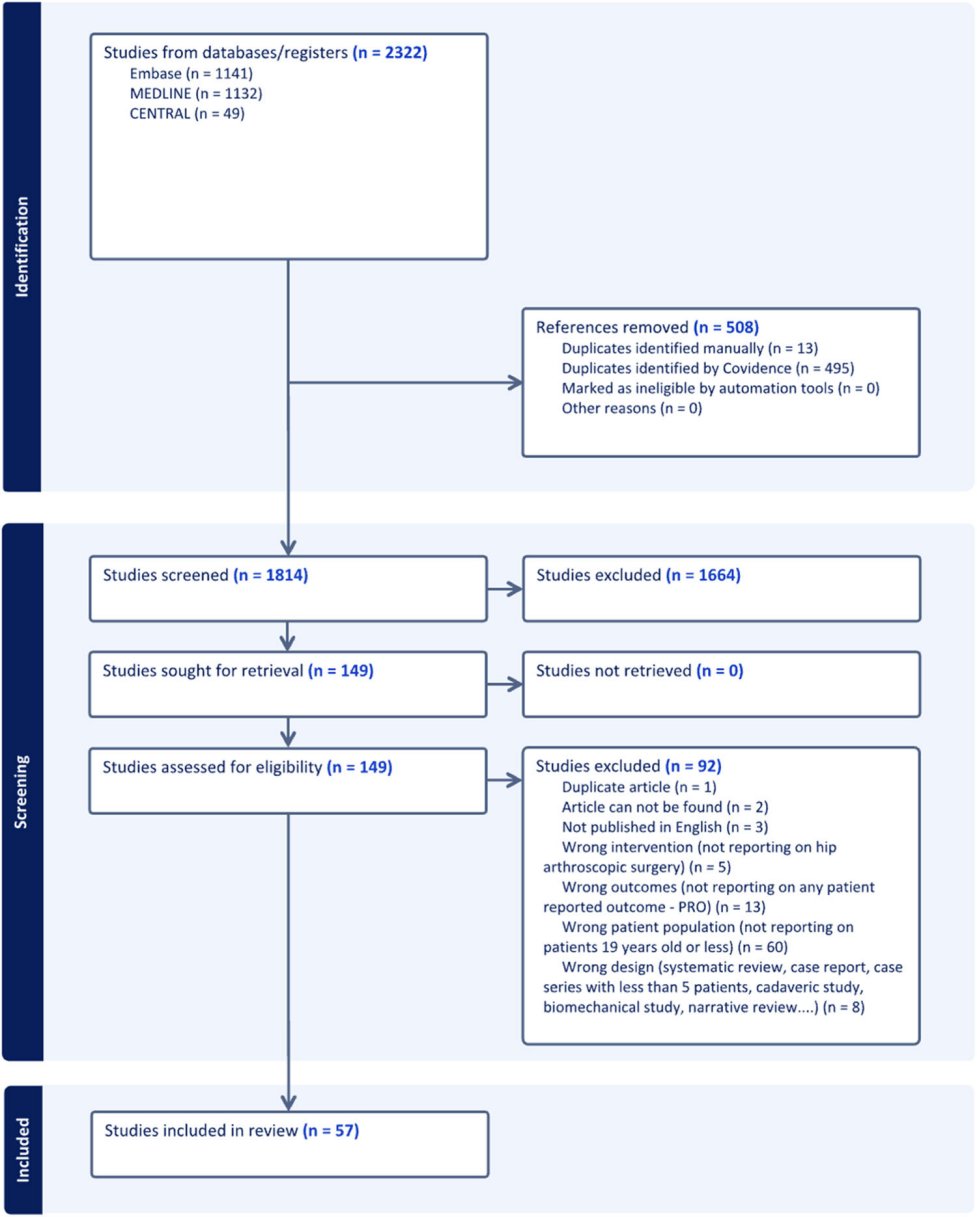
PRISMA flow diagram of screened studies from the first literature search. PRISMA, Preferred Reporting Items for Systematic Reviews and Meta‐analysis; PRO, patient‐reported outcome.

**Figure 2:**
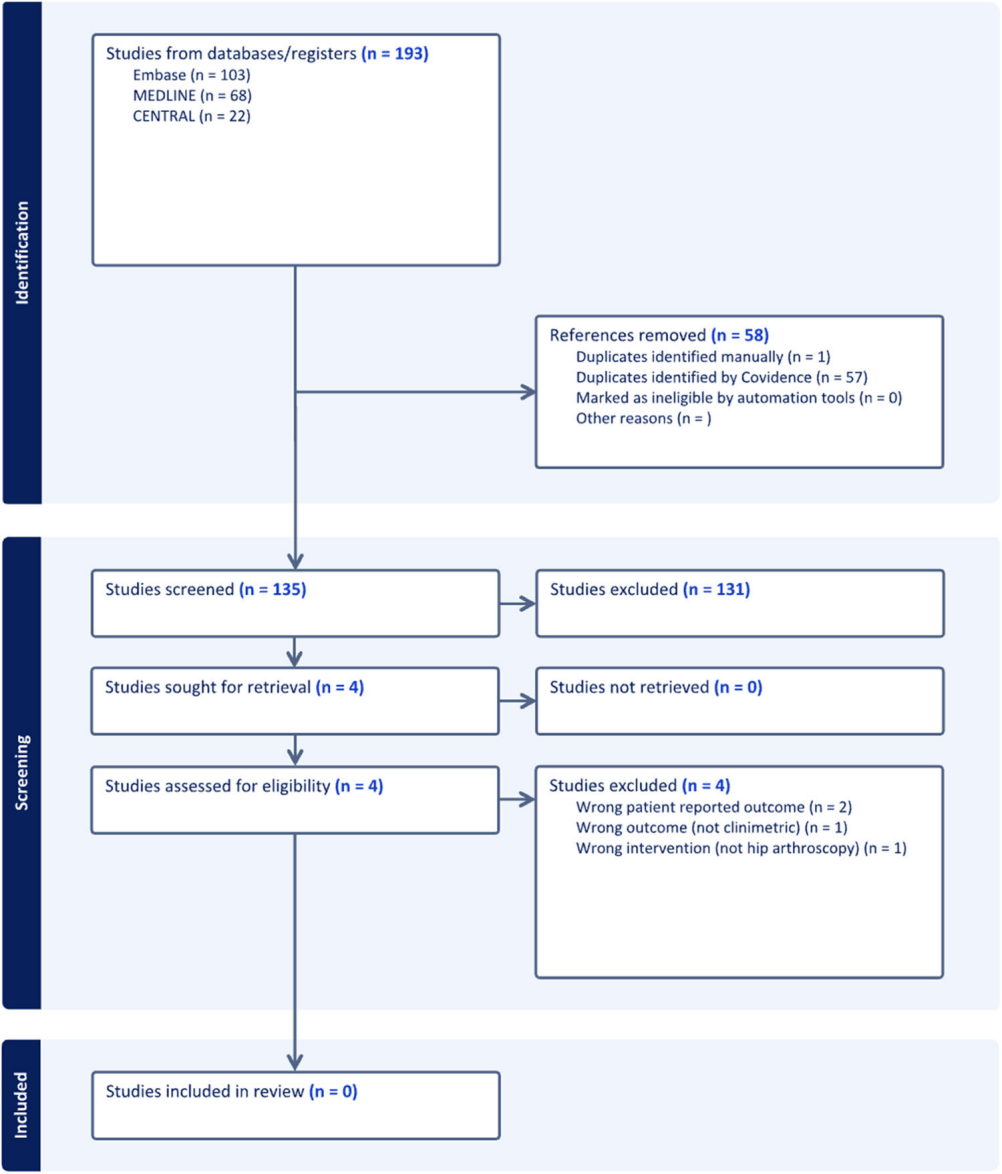
PRISMA flow diagram of screened studies from the second literature search. PRISMA, Preferred Reporting Items for Systematic Reviews and Meta‐analysis.

The first literature search identified 1814 titles potentially relevant to the PROs of interest (Figure 1). Of these, 57 articles fitting the inclusion criteria were analyzed to identify the different hip‐specific and non‐specific PROs used. The second search identified the clinimetric properties of the relevant studies identified from the first search, identifying 135 relevant titles, four of those eligible for full‐text review, but none eligible for final inclusion (Figure 2).

### Study characteristics and patient demographics

The mean age of patients was 15.9 years (range: 5.2–18.8) in the 3015 hips evaluated in 53 studies (Table 1). The mean per cent of female patients was 62.3% (range: 12.2–90.9) across studies reporting this demographic. The mean follow‐up time was 40.0 months (range: 2.4–144.0). This study identified a wide range of paediatric hip pathologies, with over seven different conditions treated via arthroscopy. The three most common pathologies assessed were FAIS with labral tear (*n* = 41; 8%), SCFE (*n* = 3; 6%) and hip dysplasia/borderline dysplasia (*n* = 2; 4%).

**Table 1:** Summary study and patient characteristics for all studies by subgroup.

	Age, years			Per cent female			Follow‐up, months		
	N.S.	Hips*, *n*	Mean (range), years	N.S.	Hips*, *n*	Mean (range), %	N.S.	Hips*, *n*	Mean (range), months
All studies	53	3015	15.9 (5.2–18.8)	47	2894	62.3 (12.2–90.9)	49	2928	40.0 (2.4–144.0)
Publication type									
Manuscript	44	2366	15.8 (5.2–17.6)	41	2322	63.1 (12.2–90.9)	42	2323	45.2 (12.0–144.0)
Presentation	9	649	16.2 (12.4–18.8)	6	572	59.4 (40.0–90.9)	7	605	20.2 (2.4–40.8)
Pathology									
Femoroacetabular impingement and/or labral tear	41	2824	16.2 (14.7–18.8)	38	2749	63.1 (12.2–90.9)	38	2749	40.1 (4.8–144)
Hip dysplasia or borderline dysplasia	2	45	15.75 (15.7–15.8)	1	21	81.0	2	45	14.7 (2.4–28.6)
Hip septic arthritis	2	27	5.6 (5.2–6.2)	1	15	40.0	1	15	26.1
Tuberculosis of the hip joint	2	44	10.3 (10.2–10.5)	2	44	36.4 (36.4–36.4)	2	44	38.1 (31.2–45.0)
Multiple indications	1	34	14.8	1	34	42.0	‐	‐	‐
Legg‐Calve‐Perthes	1	10	12.7	‐	‐	‐	1	10	55.4
Slipped capital femoral epiphysis	3	26	13.7 (12.0–15.8)	3	26	57.7 (45.5–80.0)	3	26	60.3 (26.0–88.9)
Traumatic hip dislocation	1	5	12.4	1	5	40.0	1	5	24.8

*Note*: *If number of hip not available then number of patients used as weight.

Abbreviation: N.S., number of studies.

### PROs

This study identified 10 hip‐specific PRO instruments and 5 non‐specific PRO instruments (Table 2). An average of 2.8 (SD = 1.4) individual PRO per study, with 31 studies (54%) reporting on three different PROs or more.

**Table 2:** List of included patient‐reported outcome (PRO) instruments found in paediatric hip arthroscopy literature.

Included PRO instruments	
Hip‐specific PRO instruments (*n* = 10)	Harris Hip Score
	Modified Harris Hip Score
	Western Ontario and McMaster Universities Osteoarthritis Index
	International Hip Outcome Tool‐12
	International Hip Outcome Tool‐33
	Hip Outcome Score – Sport‐Specific Subscale
	Hip Outcome Score – Activities of Daily Living Subscale
	Non‐Arthritic Hip Score
	Copenhagen Hip and Groin Outcome Score
	Hip Disability and Osteoarthritis Outcome Score
Non‐hip‐specific PRO instruments (*n* = 5)	Tegner Activity Scale
	12‐Item Short Form Survey
	Visual Analogue Scale for Pain
	12‐Item Veterans‐Rand
	University of California, Los Angeles Activity Score

**Figure 3:**
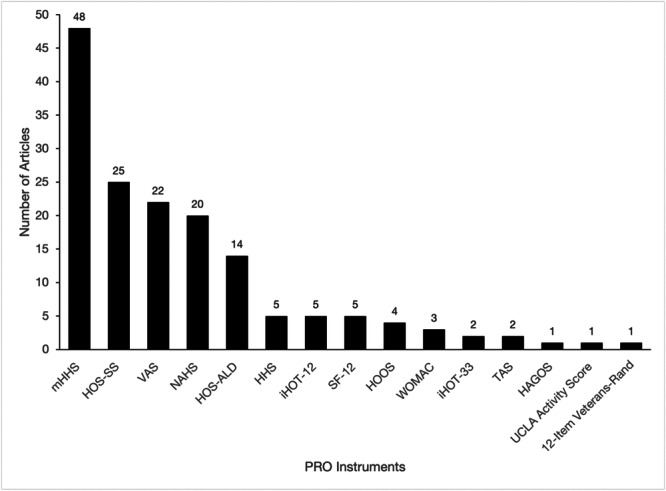
Frequency of PRO tools used in paediatric hip arthroscopy studies. HAGOS, Copenhagen Hip and Groin Outcome Score; HHS, Harris Hip Score; HOOS, Hip disability and Osteoarthritis Outcome Score; HOS‐ALD, Hip Outcome Score–Activities of Daily Living Subscale; HOS‐SS, Hip Outcome Score–Sport‐Specific Subscale; iHOT‐12, International Hip Outcome Tool‐12; iHOT‐33, International Hip Outcome Tool‐33; mHHS, modified Harris Hip Score; NAHS, Non‐Arthritic Hip Score; PRO, patient‐reported outcome; SF‐12, 12‐Item Short Form Survey; TAS, Tegner Activity Scale; UCLA Activity Score, University of California, Los Angeles Activity Score; VAS, Visual Analogue Scale for Pain; WOMAC, Western Ontario and McMaster Universities Osteoarthritis Index.

**Figure 4:**
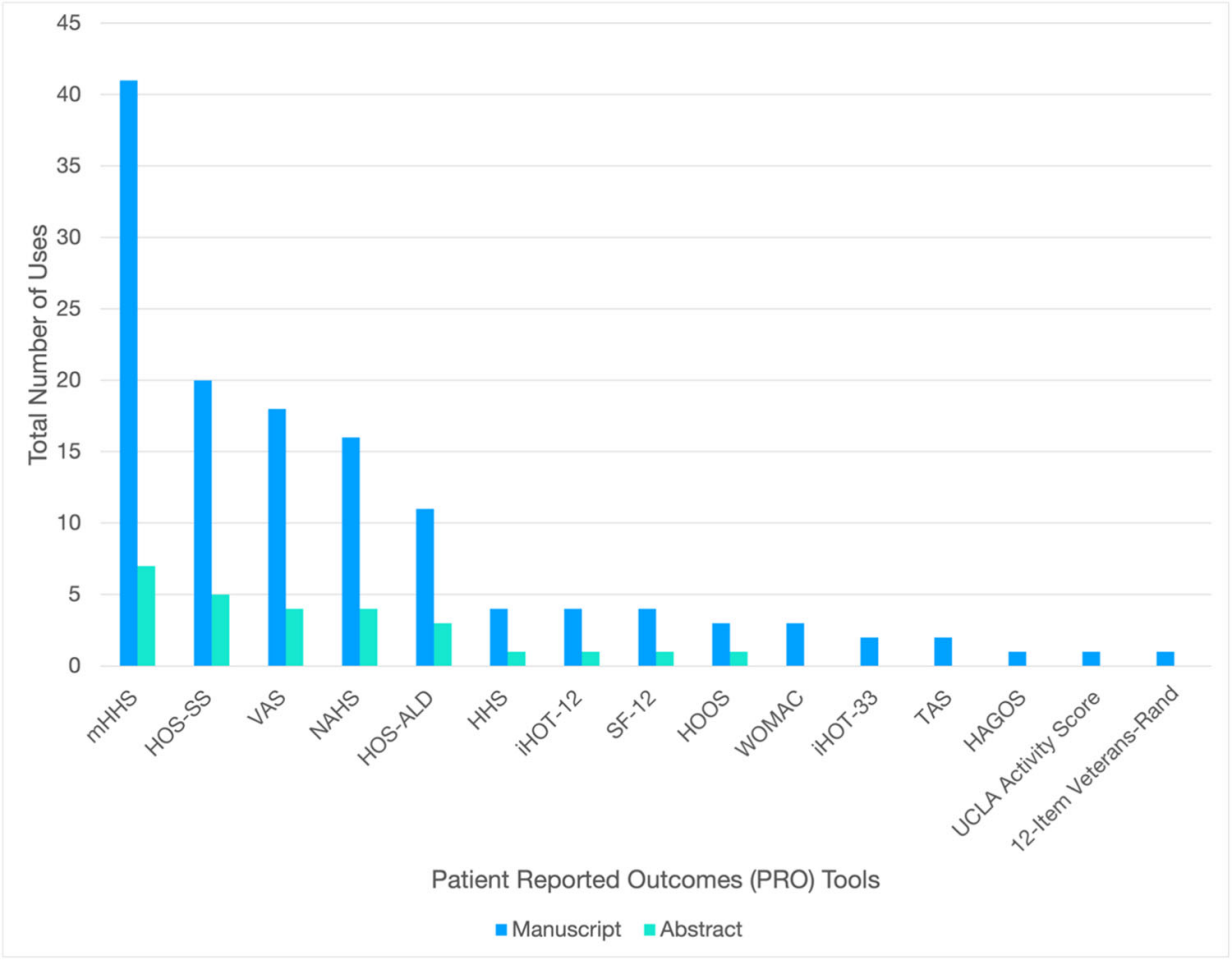
Frequency of PRO tools by publication type. HAGOS, Copenhagen Hip and Groin Outcome Score; Hip Outcome Score–Activities of Daily Living Subscale; HHS, Harris Hip Score; HOOS, Hip Disability and Osteoarthritis Outcome Score; iHOT‐12, International Hip Outcome Tool‐12; iHOT‐33, International Hip Outcome Tool‐33; NAHS, Non‐Arthritic Hip Score; SF‐12, 12‐Item Short Form Survey; TAS, Tegner Activity Scale; VAS, Visual Analogue Scale for Pain; WOMAC, Western Ontario and McMaster Universities Osteoarthritis Index; UCLA Activity Score, University of California, Los Angeles Activity Score; mHHS, Modified Harris Hip Score; HOS‐SS, Hip Outcome Score–Sport‐Specific Subscale.

The most commonly reported hip‐specific PRO was the modified Harris Hip Score (mHHS) (*n* = 48), followed by the Hip Outcome Score–Sport Specific scale (HOS‐SS) (*n* = 25) and the Non‐Arthritic Hip Score (NAHS) (*n* = 20) (Figure 3). This trend persisted when included studies were stratified by publication type, with a relatively equal distribution of outcomes across presentation abstracts and manuscripts (Figure 4).

**Figure 5:**
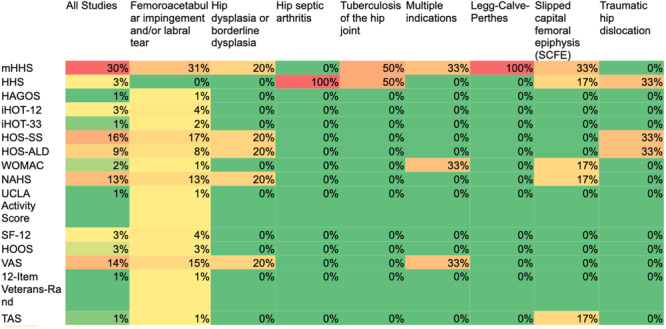
Frequency of PRO tools utilized by pathology assessed. HAGOS, Copenhagen Hip and Groin Outcome Score; HHS, Harris Hip Score; HOOS, Hip disability and Osteoarthritis Outcome Score; HOS‐ALD, Hip Outcome Score – Activities of Daily Living Subscale; HOS‐SS, Hip Outcome Score – Sport‐Specific Subscale; iHOT‐12, International Hip Outcome Tool‐12; iHOT‐33, International Hip Outcome Tool‐33; mHHS, modified Harris Hip Score; NAHS, Non‐Arthritic Hip Score; PRO, patient‐reported outcome; SF‐12, 12‐Item Short Form Survey; TAS, Tegner Activity Scale; UCLA Activity Score, University of California, Los Angeles Activity Score; VAS, Visual Analogue Scale for Pain; WOMAC, Western Ontario and McMaster Universities Osteoarthritis Index.

There was a wide distribution in the frequency of the utilization of different hip‐specific PROs by the pathology assessed (Figure 5). Notably, the majority of studies focusing on paediatric FAIS and labral tear reported on mHHS (31%) and 33% of SCFE studies also used the mHHS. In FAIS studies, the least commonly used hip‐specific PRO instruments were the Copenhagen Hip and Groin Outcome Score (HAGOS) and International Hip Outcome Tool‐33 (iHOT‐33), each used in only 1% of studies. For hip dysplasia, the use of hip‐specific PRO tools was equally distributed, with the mHHS, HOS‐SS, and Hip Outcome Score–Activities of Daily Living (HOS‐ALD) each being used in 20% of studies. The remaining pathologies were assessed by a limited number of studies preventing any substantial analysis of trends.

### Temporal trends

**Figure 6:**
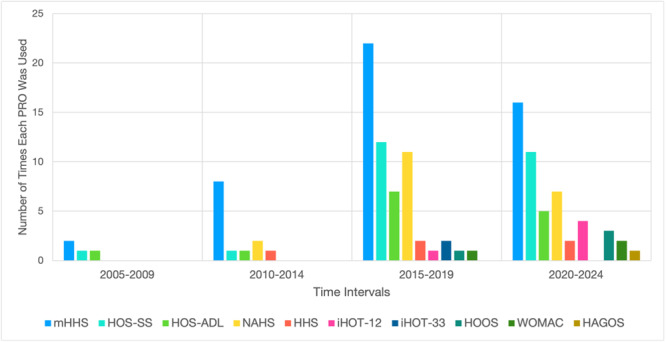
Frequency of patient‐reported outcome (PRO) tools in studies published by 5‐year intervals. HAGOS, Copenhagen Hip and Groin Outcome Score; HOOS, Hip Disability and Osteoarthritis Outcome Score; HHS, Harris Hip Score; HOS‐ALD, Hip Outcome Score–Activities of Daily Living Subscale; HOS‐SS, Hip Outcome Score–Sport‐Specific Subscale; iHOT‐12, International Hip Outcome Tool‐12; iHOT‐33, International Hip Outcome Tool‐33; mHHS, Modified Harris Hip Score; NAHS, Non‐Arthritic Hip Score; WOMAC, Western Ontario and McMaster Universities Osteoarthritis Index.

Between 2005 and 2024, there was an increasing variety of hip‐specific PROs, with seven additional tools introduced after 2009. From 2005 to 2009, only three hip‐specific PROs were reported, with the mHHS being the most commonly used (*n* = 2, 50%). Between 2010 and 2014, five hip‐specific PROs were reported, with the mHHS again most commonly used (*n* = 8, 53%). From 2015 to 2019, several established hip‐specific PROs such as the WOMAC (*n* = 1, 2%), International Hip Outcome Tool‐12 (iHOT‐12) (*n* = 1, 2%), iHOT‐33 (*n* = 2, 3%), were used in paediatric hip arthroscopy studies for the first time. During this period, the most frequently used hip‐specific PRO tools were the mHHS (*n* = 22, 29%), HOS‐SS (*n* = 12, 16%), followed by the HOS‐ALD (*n* = 7). The 2020 to 2024 period exhibited the most heterogeneity, featuring a wide distribution of studies using nine different PROs, including the first use of the Hip disability and Osteoarthritis Outcome Score (HOOS) (*n* = 3, 6%) and the HAGOS tools (*n* = 1, 2%). The mHHS (*n* = 16, 25%) and HOS‐SS (*n* = 11, 17%) remained most commonly reported during this time (Figure 6).

### Methodological quality of included studies

Among full‐text articles, the average MINORS score was 17.7 out of 24, or moderate quality, for comparative studies, and 10.5 out of 16, moderate quality, for non‐comparative studies (Supporting Information S1: Table S2). Among abstracts, the average MINORS score was 13.0 out of 24, moderate quality, for one comparative study and 8.9 out of 16, or low quality, for non‐comparative studies.

### Clinimetric evidence

The second search did not yield any studies assessing the validity or clinimetric properties of the identified PRO instruments currently used in paediatric populations.

## DISCUSSION

This review serves as an important update on paediatric hip arthroscopy, providing the first comprehensive evaluation of PROs since 2015 [6]. Notably, the heterogeneity of hip‐specific PRO use has increased during this period, with several new tools introduced and utilized since 2015. We identified ten hip‐specific PRO instruments used across various paediatric hip pathologies, including FAIS and labral tears, SCFE and hip dysplasia, with the mHHS being the most frequently utilized. Concerningly, none of these tools have been validated in the paediatric and adolescent patient population. This reliance on unvalidated PRO instruments is reflected consistently across both presentation abstracts and peer‐reviewed manuscripts.

This study highlights the use of several PRO instruments that warrant validation in paediatric populations. Among these, the mHHS, HOS‐SS and NAHS were the most commonly used hip‐specific tools. However, the frequent use of mHHS is particularly concerning, given its design for evaluating outcomes in arthritic hip pain [12, 17]. Its design as a PRO for degenerative hip disease makes it inherently unsuitable for evaluating joint‐preserving surgeries in younger patients with conditions such as FAIS or hip dysplasia Moreover, its focus on lower intensity activities, such as walking ability and navigating stairs, does not fully reflect the paediatric patient experience, raising concerns about its reliability. Similarly, both the HOS‐SS and NAHS, are commonly used to assess functional outcomes in younger, active populations, but validation studies for these tools predominantly involve middle‐aged subjects, and neither has been validated for paediatric use [5, 19, 21, 22]. The iHOT‐12, a newer tool for active adults, has seen increasing use in paediatric hip arthroscopy studies, although it includes items that are less appropriate for paediatric patients, such as carrying children and sexual activities [9]. Some authors and organizations, such as the North American Hip Arthroscopy Registry have adapted the tool to a 10‐item version by removing certain items [16]. However, this version remains unvalidated for children and adolescents, highlighting the need for age‐specific validation to ensure accurate and meaningful outcome assessments. Importantly, a systematic review by Suryavanshi et al. on paediatric sports PROs with a more general focus reported on finding one validated hip function score for paediatric populations, which was the WOMAC score [31]. However, the primary article used to make that conclusion only assessed the reliability and responsiveness of the WOMAC in a paediatric population with knee pain [14]. Therefore, as stated above, no hip function PRO has ever been validated for use in a paediatric population with hip pain.

An additional critical factor in assessing the validity of measurement tools, beyond demographic considerations, is the specific pathology being assessed. In this review, although a wide range of pathologies treated in paediatric hip arthroscopy was highlighted, FAIS constituted the majority of cases. Analogous to ACL tears in paediatric knee arthroscopy, whose outcomes can be evaluated with both specific and less specific PRO measures [8, 34], there is a clear need to develop and validate PROs tailored specifically for paediatric FAIS [28]. Moreover, the rise in early sports specialization has led to an increase in paediatric FAIS cases, emphasizing the current need for a validated paediatric hip‐specific PRO tool [7, 25]. As ongoing trials seek to understand the pathophysiology and the long‐term impact of this condition on young patients, the importance of targeted outcome measures in this population becomes increasingly clear [1, 4].

Furthermore, more than half of the analysed studies reported on three or more hip‐specific PROs, including individual tools such as HAGOS, HOOS and iHOT‐33 containing over 25 questions. This approach may inadvertently lead to respondent fatigue, especially in paediatric populations, potentially compromising the accuracy of responses [18]. This underscores the importance of carefully selecting PRO tools to balance comprehensiveness with feasibility, particularly in younger patients.

In addition to developing novel PROs, another promising solution is the adaptation of adult PROs through a rigorous methodology for their use in paediatric patients. Previous literature has demonstrated the effectiveness of this strategy to meet the unique needs of young orthopaedic patients. For instance, Hansen et. al created the KIDS‐KNEES‐ACL, a content‐valid PRO tool for children with an ACL injury [11]. Through a thematic analysis of semi‐structured interviews in children, they identified 19 new items alongside probing items from the original adult PRO ‘KNEES‐ACL’, making the tool more tailored to the paediatric population [11]. Similarly, Kocher et. al developed a modified International Knee Documentation Committee Subjective Knee Form (pedi‐IKDC) to assess knee‐specific symptoms, function, and sports activity in children and adolescents, which was validated against the Child Health Questionnaire and showed acceptable psychometric performance [20]. While promising, this method poses several challenges, including significant variability in activity levels and comprehension among children, which must be considered when designing questions that accurately reflect this patient population's experiences [2]. Nevertheless, careful adaptation using a systematic approach could improve the accuracy and relevance of PRO tools for paediatric hip care and ensure adequate measurement of outcomes.

Until an age‐specific PRO tool is developed and validated, the authors acknowledge that internally adapting adult PRO tools for paediatric use may remain a necessary interim solution. This adaptation could involve removing age‐inappropriate items and modifying the vocabulary to suit younger populations. To ensure the maximized validity of this temporary approach, it is essential to select PRO tools originally developed for age groups and contexts closely aligned with paediatric needs, thereby excluding tools like the mHHS and HHS. The reporting of any unvalidated or non‐age‐specific PROs used in the manuscript's limitations is encouraged for transparency. Furthermore, including the adapted version in the appendix is recommended to enhance transparency and support the future development of age‐specific PRO instruments.

### Strengths and limitations

Findings from this study should be interpreted with caution due to the following limitations. First, while a wide variety of hip pathologies were assessed, the analysis was predominantly focused on FAIS. This concentration may limit the generalizability of the findings to other conditions that were less frequently represented. Second, our study defined the paediatric population as patients 19 years or younger, which is a broad age range that may obscure differences between younger children and older adolescents with distinct clinical needs [30]. Furthermore, only English‐language publications were included, which, while covering most relevant literature, might exclude insights from non‐English studies. As hip arthroscopy use expands internationally, translation and cross‐cultural validation of paediatric PRO tools will be a crucial and challenging task for future research [10]. Despite these limitations, the study offers significant strengths. It provides an updated and comprehensive overview of paediatric hip pathologies, highlights the distribution of PRO tools across different conditions, and identifies temporal trends in their use.

## CONCLUSION

The key finding of this study is the ongoing lack of validated hip‐specific PRO tools in the paediatric hip arthroscopy literature, with reliance on adult‐derived instruments. The absence of clinimetric studies and increasing heterogeneity in PRO use emphasises the need for standardized, paediatric‐specific tools. Given their limitations, tools commonly used in the literature over the years, such as the mHHS, should be avoided in paediatric populations. To address this gap, developing and validating such instruments should be prioritized to ensure accurate, age‐appropriate outcome assessment and improved care. A feasible next step is to adapt adult PRO tools for paediatric use by collaborating with paediatric orthopaedic specialists and psychometricians to ensure the tools are developmentally appropriate and accurately capture the key aspects of paediatric hip conditions.

## AUTHOR CONTRIBUTIONS

Olufemi R. Ayeni and Yoan Bourgeault‐Gagnon were involved in the study conception and design. Ayomide Michael Ade‐Conde, Brendan Amoyaw and Yoan Bourgeault‐Gagnon were responsible for study screening. Ayomide Michael Ade‐Conde, Brendan Amoyaw, Yoan Bourgeault‐Gagnon and Hassaan Abdel Khalik performed various elements of the data extraction and quality assessment. Yoan Bourgeault‐Gagnon and Hassaan Abdel Khalik performed the statistical analyses. Ayomide Michael Ade‐Conde, Brendan Amoyaw, Yoan Bourgeault‐Gagnon, Hassaan Abdel Khalik, Nicole Simunovic and Olufemi R. Ayeni were involved in drafting and reviewing the manuscript.

## CONFLICT OF INTEREST STATEMENT

Olufemi R. Ayeni is on the speaker bureau for Stryker Canada, is Tier 2 Canada Research Chair in Joint Preservation, and is President/Owner of Notch Academy. The remaining authors declare no conflicts of interest.

## ETHICS STATEMENT

No ethical approval was required for this systematic review.

## Supporting information

Supporting Information.

## Data Availability

Data are available upon reasonable request to the corresponding author.
