# Supplementary material for: Lack of validated patient‐reported outcome tools persists in paediatric and adolescent hip arthroscopy—A systematic review
Source: Knee Surg Sports Traumatol Arthrosc. 2025 Jan 29;33(5):1863–73. doi: 10.1002/ksa.12603 (PMC12022817; doi:10.1002/ksa.12603)
Supplement: Supplementary file 1 — Supporting Information. [file KSA-33-1863-s001.docx]

**Online Resource 1, Table S1 and Table S2**

| **Table S1** Search Strategies**Database Searched** | **Search String** | **Filters/Limits** | **Date Searched** | **# Of Records Identified** |
| --- | --- | --- | --- | --- |
| **1^st^ Search: Reported PROs Used in Paediatric Hip Literature** | | | | |
| OVID:   1. Ovid MEDLINE(R) ALL 1946 to March 31st, 2024 2. EBM Reviews – Cochrane Register of Controlled Trials March 2024 3. EBM Reviews – Cochrane Methodology Register 3^rd^ Quarter 2012 | ((exp Hip/ OR exp Hip Joint/ OR exp Acetabulum/ OR exp Femoracetabular Impingement/ OR exp Hip Injuries/ OR exp Hip Dislocation/ OR hip*.ti,ab. OR Acetabu*.ti,ab. OR Femoroacetabular impingement.ti,ab. OR (femoroacetabular adj2 impingement).ti,ab. OR femoro acetabular.ti,ab. OR femoral acetabular.ti,ab. OR (cam adj1 impingement).ti,ab. OR (pincer adj1 impingement).ti,ab. OR FAI.mp. OR Borderline hip dysplasia.mp. OR Hip dysplasia*.ti,ab. OR exp Legg-Calve-Perthes Disease/ OR Perthes*.ti,ab. OR exp Slipped Capital Femoral Epiphyses/ OR SCFE.ti,ab. OR Labral tear.mp.))  AND  (Arthroscop*.ti,ab. OR exp Arthroscopy/)  AND  (exp Child/ OR exp Adolescent/ OR exp Infant/ OR exp Child, Preschool/ OR exp Pediatrics/ OR Teenage*.ti,ab. OR Adolescent*.ti,ab. OR Infant*.ti,ab. OR Paediatric*.ti,ab. OR Child*.ti,ab. OR exp Infant, Newborn/) | None | March 31^st^, 2024 | MEDLINE: 1132  CENTRAL: 49 |
| EMBASE: 1974 to 2024 March 31 | ((('labral tear*':ti,ab) OR (scfe*:ti,ab) OR ('hip dysplasia*':ti,ab) OR ('slipped capital*':ti,ab) OR (perthes*:ti,ab) OR ('femoroacetabular impingement*':ti,ab) OR (hip*:ti,ab) OR ('borderline hip dysplasia*') OR ('labral tear*') OR ('slipped capital femoral epiphysis') OR 'perthes disease'/exp OR 'femoroacetabular impingement'/exp OR 'hip dysplasia'/exp OR 'hip injury'/exp OR 'hip dislocation'/exp OR 'hip disease'/exp OR ('hip'/exp OR hip)))  AND  ((arthroscop*:ti,ab) OR ('arthroscopy'))  AND  (('newborn') OR ('pediatrics') OR (adolescent*:ti,ab) OR (infant*:ti,ab) OR (pediatric*:ti,ab) OR (teenage*:ti,ab) OR (child*:ti,ab) OR ('infant') OR ('adolescent') OR 'child'/exp)  AND [embase]/lim | Embase | March 31^st^, 2024 | EMBASE: 1142 |
| **2^nd^ Search: Clinimetric Studies on the Hip-Function PROs Used in Paediatric Populations** | | | | |
| OVID:   1. Embase 1974 to 2024 August 21 2. EBM Reviews - Cochrane Central Register of Controlled Trials July 2024 3. EBM Reviews - Cochrane Methodology Register 3rd Quarter 2012 4. Ovid MEDLINE(R) ALL 1946 to August 21, 2024 | (Modified Harris Hip Sc* or mHHS or Harris Hip Sc* or HHS or "Hip Disability and Osteoarthritis Outcome Sc*" or HOOS or NAHS or Non-Arthritic Hip Sc* or Hip Outcome Sc* or HOS or "Hip Outcome Score - Sport-Specific Subscale" or "HOS-SS" or "Hip Outcome Score - Activities of Daily Living" or "HOS-ADL" or "Western Ontario and McMaster Universities Osteoarthritis Index" or WOMAC or International Hip Outcome Tool or iHOT or "International Hip Outcome Tool-12" or "iHOT-12" or "International Hip Outcome Tool-33" or "iHOT-33" or "Copenhagen Hip and Groin Outcome Score" or HAGOS).ti,ab. AND (pedia* or paedia* or adolescent or child* or teen* or youth).ti,ab. AND (reliability or validity or valid* or responsiveness or clinimetric or "measurement properties" or psychometric* or consistency or error or reproducibility or cronbach* or interpretability or "factor analysis").ti,ab | None | August 21^st^, 2024 | MEDLINE: 103  EMBASE: 68  CENTRAL: 22 |

**Table S2** MINORS Quality Assessment

|  |  | **MINORS Assessment Scored 0-2 (0=not reported, 1=reported but inadequate, 2=reported and adequate)** | | | | | | | | | | | | |  |
| --- | --- | --- | --- | --- | --- | --- | --- | --- | --- | --- | --- | --- | --- | --- | --- |
|  |  |  |  |  |  |  |  |  |  |  | **Comparative Studies** | | | |  |
| **Study** | **Format** | **A clearly stated aim** | **Inclusion of consecutive patients** | **Prospective collection of data** | **Endpoints appropriate to the aim of the study** | **Unbiased assessment of the study endpoint** | **Follow-up period appropriate to the aim of the study** | **Loss to follow up less than 5%** | **Prospective calculation of the study size** |  | **An adequate control group** | **Contemporary groups** | **Baseline equivalence of groups** | **Adequate statistical analyses** | **Total Score** |
| Akkari 2010[1] | Full-Text | 2 | 2 | 2 | 2 | 0 | 1 | 2 | 0 |  |  |  |  |  | **11** |
| AlamKhan 2013[23] | Full-Text | 2 | 0 | 0 | 2 | 0 | 2 | 0 | 0 |  |  |  |  |  | **6** |
| Arashi 2019[2] | Full-Text | 2 | 1 | 1 | 2 | 1 | 2 | 1 | 0 |  |  |  |  |  | **10** |
| Ashberg 2018 [3] | Full-Text | 2 | 2 | 2 | 2 | 0 | 2 | 1 | 0 |  | 2 | 2 | 1 | 2 | **18** |
| Balakumar 2019[4] | Full-Text | 2 | 2 | 1 | 2 | 0 | 2 | 2 | 0 |  |  |  |  |  | **11** |
| Barastegui 2023[5] | Full-Text | 2 | 2 | 2 | 2 | 0 | 2 | 2 | 0 |  |  |  |  |  | **12** |
| Byrd 2013[50] | Abstract | 2 | 2 | 2 | 2 | 0 | 1 | 0 | 0 |  |  |  |  |  | **9** |
| Byrd 2016-1[6] | Full-Text | 2 | 2 | 2 | 2 | 0 | 2 | 0 | 1 |  |  |  |  |  | **11** |
| Byrd 2016-2[7] | Full-Text | 2 | 2 | 2 | 2 | 1 | 2 | 2 | 0 |  |  |  |  |  | **13** |
| CapurroSoler 2016[8] | Abstract | 2 | 1 | 1 | 2 | 0 | 2 | 1 | 0 |  |  |  |  |  | **9** |
| Chandrasekaran 2017[9] | Full-Text | 2 | 2 | 2 | 2 | 0 | 2 | 1 | 0 |  | 2 | 2 | 2 | 0 | **17** |
| Cooper 2022[10] | Abstract | 2 | 0 | 2 | 2 | 0 | 2 | 0 | 0 |  |  |  |  |  | **8** |
| Cvetanovich 2018[11] | Full-Text | 2 | 2 | 2 | 2 | 2 | 2 | 1 | 0 |  |  |  |  |  | **13** |
| Degen 2017[12] | Full-Text | 2 | 2 | 2 | 2 | 0 | 2 | 1 | 0 |  |  |  |  |  | **11** |
| Domb 2023-1[14] | Abstract | 2 | 2 | 2 | 2 | 0 | 2 | 0 | 0 |  |  |  |  |  | **10** |
| Domb 2023-2[13] | Abstract | 2 | 0 | 2 | 2 | 0 | 2 | 0 | 0 |  |  |  |  |  | **8** |
| Duman 2020[15] | Full-Text | 2 | 2 | 1 | 2 | 0 | 2 | 1 | 0 |  | 2 | 2 | 2 | 0 | **16** |
| Ejnisman 2011[16] | Full-Text | 2 | 2 | 2 | 2 | 0 | 2 | 0 | 0 |  |  |  |  |  | **10** |
| Evans 2016[17] | Abstract | 2 | 2 | 2 | 2 | 0 | 2 | 1 | 0 |  |  |  |  |  | **11** |
| Evans 2017[18] | Full-Text | 2 | 2 | 2 | 2 | 0 | 2 | 1 | 0 |  |  |  |  |  | **11** |
| Fabricant 2012[19] | Full-Text | 2 | 2 | 2 | 2 | 0 | 1 | 2 | 0 |  |  |  |  |  | **11** |
| Fukase 2022[20] | Full-Text | 2 | 2 | 2 | 2 | 0 | 2 | 1 | 0 |  |  |  |  |  | **11** |
| Jimenez 2022[21] | Full-Text | 2 | 2 | 2 | 2 | 0 | 2 | 1 | 0 |  |  |  |  |  | **11** |
| Kanatli 2019[22] | Full-Text | 2 | 0 | 1 | 2 | 0 | 2 | 2 | 0 |  |  |  |  |  | **9** |
| Kivlan 2021[24] | Full-Text | 2 | 0 | 0 | 2 | 0 | 2 | 0 | 0 |  | 2 | 2 | 2 | 2 | **14** |
| Kocher 2005[25] | Full-Text | 2 | 2 | 2 | 2 | 0 | 1 | 2 | 0 |  |  |  |  |  | **11** |
| Larson 2019[26] | Full-Text | 2 | 2 | 2 | 2 | 0 | 2 | 0 | 0 |  |  |  |  |  | **10** |
| Lee 2022[27] | Full-Text | 2 | 2 | 2 | 2 | 0 | 2 | 1 | 2 |  | 2 | 2 | 1 | 2 | **20** |
| Lim 2020[28] | Full-Text | 2 | 2 | 1 | 2 | 0 | 2 | 0 | 0 |  |  |  |  |  | **9** |
| Litrenta 2019[31] | Full-Text | 2 | 2 | 2 | 2 | 0 | 2 | 0 | 0 |  | 2 | 2 | 2 | 2 | **18** |
| Litrenta 2019[29] | Full-Text | 2 | 2 | 2 | 2 | 0 | 2 | 1 | 0 |  |  |  |  |  | **11** |
| Litrenta 2020[30] | Full-Text | 2 | 2 | 2 | 2 | 0 | 2 | 1 | 0 |  |  |  |  |  | **11** |
| Maldonado 2023[32] | Full-Text | 2 | 2 | 2 | 2 | 0 | 2 | 1 | 0 |  | 2 | 2 | 2 | 2 | **19** |
| McConkey 2019[33] | Full-Text | 2 | 0 | 2 | 2 | 0 | 2 | 2 | 0 |  |  |  |  |  | **10** |
| Menge 2021[34] | Full-Text | 2 | 0 | 2 | 2 | 0 | 2 | 1 | 0 |  |  |  |  |  | **9** |
| Murtha 2021[35] | Full-Text | 2 | 0 | 2 | 2 | 0 | 1 | 1 | 0 |  | 1 | 2 | 2 | 2 | **15** |
| Newman 2015[37] | Full-Text | 2 | 2 | 2 | 2 | 0 | 2 | 0 | 0 |  |  |  |  |  | **10** |
| Newman 2016[36] | Full-Text | 2 | 2 | 2 | 2 | 0 | 2 | 2 | 2 |  | 2 | 2 | 1 | 2 | **21** |
| Nwachukwu 2017[38] | Full-Text | 2 | 2 | 2 | 2 | 0 | 1 | 1 | 0 |  | 2 | 2 | 1 | 2 | **17** |
| O'Donnell 2012[39] | Full-Text | 2 | 2 | 2 | 2 | 0 | 1 | 2 | 0 |  |  |  |  |  | **11** |
| Perets 2016[40] | Abstract | 2 | 0 | 0 | 2 | 0 | 2 | 0 | 0 |  | 2 | 2 | 2 | 1 | **13** |
| Perets 2017[41] | Full-Text | 2 | 2 | 2 | 2 | 1 |  | 0 | 0 |  |  |  |  |  | **9** |
| Philippon 2008[43] | Full-Text | 2 | 2 | 2 | 2 | 1 | 1 | 0 | 0 |  |  |  |  |  | **10** |
| Philippon 2012[42] | Full-Text | 2 | 2 | 2 | 2 | 0 | 2 | 2 | 0 |  |  |  |  |  | **12** |
| Rahm 2019[44] | Full-Text | 2 | 2 | 1 | 2 | 1 | 2 | 1 | 0 |  |  |  |  |  | **11** |
| Ross 2017[45] | Full-Text | 2 | 0 | 2 | 2 | 0 | 1 | 0 | 0 |  |  |  |  |  | **7** |
| Rudd 2013[46] | Full-Text | 2 | 2 | 2 | 2 | 0 | 1 | 1 | 0 |  | 2 | 2 | 2 | 2 | **18** |
| Ruzbarsky 2023[47] | Abstract | 2 | 0 | 0 | 2 | 0 | 2 | 1 | 0 |  |  |  |  |  | **7** |
| Sanpera 2016[48] | Full-Text | 2 | 2 | 1 | 1 | 1 | 2 | 2 | 0 |  |  |  |  |  | **11** |
| Serbin 2022[49] | Full-Text | 2 | 2 | 2 | 2 | 0 | 2 | 0 | 0 |  | 2 | 2 | 2 | 2 | **18** |
| Tiwari 2015[51] | Full-Text | 2 | 2 | 1 | 2 | 0 | 2 | 0 | 0 |  | 2 | 2 | 2 | 2 | **17** |
| Tran 2013[52] | Full-Text | 2 | 2 | 2 | 2 | 0 | 1 | 2 | 1 |  | 2 | 2 | 2 | 2 | **20** |
| Willimon 2021[53] | Abstract | 2 | 2 | 1 | 2 | 0 | 1 | 1 | 0 |  |  |  |  |  | **9** |
| Winge 2021[54] | Full-Text | 2 | 2 | 2 | 2 | 0 | 2 | 2 | 0 |  |  |  |  |  | **12** |
| Wyles 2017[55] | Full-Text | 2 | 2 | 2 | 2 | 0 | 2 | 1 | 1 |  |  |  |  |  | **12** |
| Yen 2024[56] | Full-Text | 2 | 0 | 2 | 2 | 0 | 1 | 1 | 0 |  |  |  |  |  | **8** |
| Zogby 2021[57] | Full-Text | 2 | 0 | 2 | 2 | 1 | 2 | 1 | 1 |  | 2 | 2 | 1 | 2 | **18** |

**REFERENCES**

1. Akkari M, Santili C, Braga SR, Polesello GC (2010) Trapezoidal bony correction of the femoral neck in the treatment of severe acute-on-chronic slipped capital femoral epiphysis. Arthrosc J Arthrosc Relat Surg Off Publ Arthrosc Assoc N Am Int Arthrosc Assoc 26(11):1489–1495

2. Arashi T, Murata Y, Utsunomiya H, Kanezaki S, Suzuki H, Sakai A, Uchida S (2019) Higher risk of cam regrowth in adolescents undergoing arthroscopic femoroacetabular impingement correction: a retrospective comparison of 33 adolescent and 74 adults. Acta Orthop 90(6):547–553

3. Ashberg L, Walsh JP, Yuen LC, Perets I, Chaharbakhshi EO, Domb BG (2018) Outcomes of Hip Arthroscopy in Adolescents: A Comparison of Acute Versus Chronic Presentation. Two-Year Minimum Follow-up. J Pediatr Orthop 38(2):e50–e56

4. Balakumar B, Flatt E, Madan S (2019) Moderate and severe SCFE (Slipped Capital Femoral Epiphysis) arthroscopic osteoplasty vs open neck osteotomy-a retrospective analysis of results. Int Orthop 43(10):2375–2382

5. Barastegui D, Seijas R, Alentorn-Geli E, Ferré-Aniorte A, Laiz P, Cugat R (2022) Hip arthroscopy is a successful treatment for femoroacetabular impingement in under-16 competitive football players: a prospective study with minimum 2-year follow-up. Arch Orthop Trauma Surg 143(5):2641–2646

6. Byrd JWT, Jones KS, Gwathmey FW (2016) Femoroacetabular Impingement in Adolescent Athletes: Outcomes of Arthroscopic Management. Am J Sports Med 44(8):2106–2111

7. Byrd JWT, Jones KS, Gwathmey FW (2016) Arthroscopic Management of Femoroacetabular Impingement in Adolescents. Arthrosc J Arthrosc Relat Surg Off Publ Arthrosc Assoc N Am Int Arthrosc Assoc 32(9):1800–1806

8. Capurro Soler B, Dantas P, Más Martínez J, León García A, Marques López F, Tey Pons M (2016) Hip arthroscopy for paediatric and adolescent pathologies. Indications and outcomes. HIP Int 26((Capurro Soler B.; León García A.; Marques López F.; Tey Pons M.) Hospital Del Mar-Parc de Salut Mar, Barcelona, Spain(Dantas P.) Hospital CUF Descobertas, Lisboa, Portugal(Más Martínez J.) Clínica Vistahermosa, Alicante, Spain(Tey Pons M.) ICATME, Hospit):S21

9. Chandrasekaran S, Darwish N, Chaharbakhshi EO, Lodhia P, Suarez-Ahedo C, Domb BG (2017) Arthroscopic Treatment of Labral Tears of the Hip in Adolescents: Patterns of Clinical Presentation, Intra-articular Derangements, Radiological Associations and Minimum 2-Year Outcomes. Arthrosc J Arthrosc Relat Surg 33(7):1341–1351

10. Cooper S, Johnson B, Youngman T, Wilson P, Sucato D, Podeszwa D, Ellis H, Serbin P (2022) Predictors of Re-Operation in Adolescents Undergoing Hip Preservation SUrgery for Femoroacetabular Impingement. Orthop J Sports MedDOI: 10.1177/2325967121S00586

11. Cvetanovich GL, Weber AE, Kuhns BD, Hannon CP, D’Souza D, Harris J, Mather RC, Nho SJ (2018) Clinically Meaningful Improvements After Hip Arthroscopy for Femoroacetabular Impingement in Adolescent and Young Adult Patients Regardless of Gender. J Pediatr Orthop 38(9):465–470

12. Degen RM, Mayer SW, Fields KG, Coleman SH, Kelly BT, Nawabi DH (2017) Functional Outcomes and Cam Recurrence After Arthroscopic Treatment of Femoroacetabular Impingement in Adolescents. Arthrosc J Arthrosc Relat Surg 33(7):1361–1369

13. Domb BG, Maldonado D, Kufta AY, Krych AJ, Levy BA, Okoroha KR, Gonzalez-Carta K (2023) Primary Hip Arthroscopy For Femoroacetabular Impingement Syndrome In Adolescents Improves Outcomes And Clinical Benefit Achievement Rates At Short-Term Follow-Up. A Multi-Center Analysis.

14. Domb BG, Maldonado DR, Lee M, Jimenez AE, Owens JS (2023) Primary Acetabular Labral Reconstruction In Adolescents, In the Rare Scenario of Irreparable Labral Tears, Resulted In Comparable Improvement, Clinical Benefit, and Revision Surgeries Rate to a Primary Labral Repair Benchmark Group.

15. Duman S, Camurcu Y, Ucpunar H, Çöbden A, Karahan N, Sofu H (2020) Arthroscopic Treatment of Acute Septic Arthritis of the Hip Joint in Pediatric Patients Aged 10 Years or Younger. Arthrosc J Arthrosc Relat Surg 36(2):464–472

16. Ejnisman L, Briggs KK, Lertwanich P, Philippon MJ (2011) Outcomes following hip arthroscopy in the youth athlete. Arthrosc - J Arthrosc Relat Surg 27(10):e110

17. Evans P, Redmond J, Hammarstedt J, Liu Y, Chaharbakhshi E, Domb B, Perets I (2016) Arthroscopic capsular plication for the treatment of borderline dysplasia of the hip in the adolescent population: A cohort study with minimum two-year follow-up. HIP Int 26((Evans P.; Redmond J.; Hammarstedt J.; Liu Y.; Chaharbakhshi E.; Perets I.) American Hip Institute, Westmont, United States(Domb B.) Hinsdale Orthopaedics, American Hip Institute, Westmont, United States):S21–S22

18. Evans PT, Redmond JM, Hammarstedt JE, Liu Y, Chaharbakhshi EO, Domb BG (2017) Arthroscopic Treatment of Hip Pain in Adolescent Patients With Borderline Dysplasia of the Hip: Minimum 2-Year Follow-Up. Arthrosc J Arthrosc Relat Surg 33(8):1530–1536

19. Fabricant PD, Heyworth BE, Kelly BT (2012) Hip arthroscopy improves symptoms associated with FAI in selected adolescent athletes. Clin Orthop 470(1):261–269

20. Fukase N, Murata Y, Pierpoint LA, Soares RW, Arner JW, Ruzbarsky JJ, Quinn PM, Philippon MJ (2022) Outcomes and Survivorship at a Median of 8.9 Years Following Hip Arthroscopy in Adolescents with Femoroacetabular Impingement: A Matched Comparative Study with Adults. J Bone Jt Surg 104(10):902–909

21. Jimenez AE, Glein RM, Owens JS, Lee MS, Maldonado DR, Saks BR, Lall AC, Domb BG (2022) Predictors of Achieving the Patient Acceptable Symptomatic State at Minimum 5-Year Follow-up Following Primary Hip Arthroscopy in the Adolescent Athlete. J Pediatr Orthop 42(3):e277–e284

22. Kanatli U, Ayanoglu T, Ozer M, Ataoglu MB, Cetinkaya M (2019) Hip arthroscopy for Legg-Calvè-Perthes disease in paediatric population. Acta Orthop Traumatol Turc 53(3):203–208

23. Khan SA (2013) Hip Arthroscopy in Children with Tuberculosis- Experience from the Developing World. Arthrosc J Arthrosc Relat Surg 29(12):e202

24. Kivlan B, Martin RR, Carreira D, Christoforetti J, Wolff A, Nho S, Salvo J, Matsuda D, Van Thiel G, Stubbs A, Suri M (2021) Multi-center Analysis of Sports-Related Outcomes of Adolescents following Hip Arthroscopy. Arthrosc J Arthrosc Relat Surg 37(1):e65–e66

25. Kocher MS, Kim Y-J, Millis MB, Mandiga R, Siparsky P, Micheli LJ, Kasser JR (2005) Hip arthroscopy in children and adolescents. J Pediatr Orthop 25(5):680–686

26. Larson CM, McGaver RS, Collette NR, Giveans MR, Ross JR, Bedi A, Nepple JJ (2019) Arthroscopic Surgery for Femoroacetabular Impingement in Skeletally Immature Athletes: Radiographic and Clinical Analysis. Arthrosc J Arthrosc Relat Surg 35(6):1819–1825

27. Lee MS, Paraschos OA, Jimenez AE, Owens JS, Maldonado DR, Domb BG (2022) 5-Year Arthroscopy-Free Survivorship and Outcomes of Adolescents Undergoing Primary Hip Arthroscopy: A Comparison Between Traumatic and Atraumatic Injuries. Am J Sports Med 50(10):2613–2621

28. Lim C, Cho T-J, Shin CH, Choi IH, Yoo WJ (2020) Functional Outcomes of Hip Arthroscopy for Pediatric and Adolescent Hip Disorders. Clin Orthop Surg 12(1):94–99

29. Litrenta J, Mu B, Chen AW, Ortiz-Declet V, Perets I, Domb BG (2019) Radiographic and Clinical Outcomes of Adolescents With Acetabular Retroversion Treated Arthroscopically. J Pediatr Orthop 39(10):510–515

30. Litrenta J, Mu BH, Ortiz-Declet V, Chen AW, Perets I, Wojnowski NM, Domb BG (2020) Hip Arthroscopy Successfully Treats Femoroacetabular Impingement in Adolescent Athletes. J Pediatr Orthop 40(3):e156–e160

31. Litrenta JM, Mu BH, Chen AW, Perets I, Ortiz-Declet V, Domb BG (2019) Arthroscopic Labral Treatment in Adolescents: Clinical Outcomes With Minimum 5-Year Follow-up. Am J Sports Med 47(4):870–875

32. Maldonado DR, Kufta AY, Krych AJ, Levy BA, Okoroha KR, Gonzalez-Carta K, Domb BG (2023) Primary Hip Arthroscopy for Femoroacetabular Impingement Syndrome in Adolescents Improves Outcomes and Clinical Benefit Achievement Rates at Short-Term Follow-Up: A Multicenter Analysis. Arthrosc J Arthrosc Relat Surg 39(5):1211–1219

33. McConkey MO, Chadayammuri V, Garabekyan T, Mayer SW, Kraeutler MJ, Mei-Dan O (2019) Simultaneous Bilateral Hip Arthroscopy in Adolescent Athletes With Symptomatic Femoroacetabular Impingement. J Pediatr Orthop 39(4):193–197

34. Menge TJ, Briggs KK, Rahl MD, Philippon MJ (2021) Hip Arthroscopy for Femoroacetabular Impingement in Adolescents: 10-Year Patient-Reported Outcomes. Am J Sports Med 49(1):76–81

35. Murtha AS, Bomar JD, Johnson KP, Upasani VV, Pennock AT (2021) Acetabular labral tears in the adolescent athlete: results of a graduated management protocol from therapy to arthroscopy. J Pediatr Orthop B 30(6):549–555

36. Newman JT, Briggs KK, McNamara SC, Philippon MJ (2016) Outcomes After Revision Hip Arthroscopic Surgery in Adolescent Patients Compared With a Matched Cohort Undergoing Primary Arthroscopic Surgery. Am J Sports Med 44(12):3063–3069

37. Newman JT, Philippon MJ, Saroki A, Briggs KK (2015) Revision Hip Arthroscopy in the Youth Athlete. Orthop J Sports Med 3(7_suppl2):2325967115S00138

38. Nwachukwu BU, Chang B, Kahlenberg CA, Fields K, Nawabi DH, Kelly BT, Ranawat AS (2017) Arthroscopic Treatment of Femoroacetabular Impingement in Adolescents Provides Clinically Significant Outcome Improvement. Arthrosc J Arthrosc Relat Surg 33(10):1812–1818

39. O’Donnell JM, Pritchard M, Tran P (2012) The outcome of arthroscopic treatment for cam type femoroacetabular impingement in adolescents. Arthrosc - J Arthrosc Relat Surg 28(6):e54-55

40. Perets I, Gupta A, Chaharbakhshi E, Hartigan D, Ashberg L, Domb B (2016) Does the femoral cam lesion regrow after arthroscopic femoroplasty in the skeletally immature patient with an open femoral head physis? Radiographic review and clinical outcomes with minimum two-year follow-up. HIP Int 26((Perets I.; Gupta A.; Chaharbakhshi E.; Hartigan D.; Ashberg L.) American Hip Institute, Westmont, United States(Domb B.) Hinsdale Orthopaedics, American Hip Institute, Westmont, United States):S33–S34

41. Perets I, Gupta A, Chaharbakhshi EO, Ashberg L, Hartigan DE, Close MR, Domb BG (2017) Does Bony Regrowth Occur After Arthroscopic Femoroplasty in a Group of Young Adolescents? Arthrosc J Arthrosc Relat Surg 33(5):988–995

42. Philippon MJ, Ejnisman L, Ellis HB, Briggs KK (2012) Outcomes 2 to 5 Years Following Hip Arthroscopy for Femoroacetabular Impingement in the Patient Aged 11 to 16 Years. Arthrosc J Arthrosc Relat Surg 28(9):1255–1261

43. Philippon MJ, Yen Y-M, Briggs KK, Kuppersmith DA, Maxwell RB (2008) Early outcomes after hip arthroscopy for femoroacetabular impingement in the athletic adolescent patient: a preliminary report. J Pediatr Orthop 28(7):705–710

44. Rahm S, Jud L, Jungwirth-Weinberger A, Sutter R, Zingg P (2019) Mid term results after in situ pinning and hip arthroscopy for mild SCFE: A minium 5-year follow-up. Swiss Med Wkly 149((Rahm S.; Jud L.; Jungwirth-Weinberger A.; Sutter R.; Zingg P.) Universitätsklinik Balgrist, Switzerland):12S

45. Ross JR, Stone RM, Ramos NM, Bedi A, Larson CM (2017) Surgery for Femoroacetabular Impingement in Skeletally Immature Patients: Radiographic and Clinical Analysis. Orthop J Sports Med 5(7_suppl6):2325967117S00259

46. Rudd J, Suri M, Choate W, Heinrich S, Christoforetti J (2013) Outcomes of arthroscopically treated femoroacetabular impingement in children and adolescents with slipped capital femoral epiphysis and Legg-calve-perthes disease. Arthrosc - J Arthrosc Relat Surg 29(12):e209–e210

47. Ruzbarsky JJ, Comfort SM, Fukase N, Vidal LB, Philippon MJ (2023) Timing From Symptom Onset to Hip Arthroscopy for Treatment of Femoroacetabular Impingement in Adolescent Patients.

48. Sanpera I, Raluy-Collado D, Sanpera-Iglesias J (2016) Arthroscopy for hip septic arthritis in children. Orthop Traumatol Surg Res 102(1):87–89

49. Serbin PA, Cooper S, Johnson BL, Youngman TR, Wilson PL, Sucato D, Podeszwa D, Ellis HB (2022) Predictors of Re-Operation in Adolesents Undergoing Hip Preservation Surgery for Femoroacetabular Impingement. Orthop J Sports Med 10(5_suppl2):2325967121S0045

50. Thomas Byrd JW, Jones K (2013) Arthroscopic Management of Femoroacetabular Impingement (FAI) in Adolescents. Arthrosc J Arthrosc Relat Surg 29(12):e209

51. Tiwari V, Khan SA, Kumar A, Poudel R, Kumar VS (2015) Functional improvement after hip arthroscopy in cases of active paediatric hip joint tuberculosis: a retrospective comparative study vis-à-vis conservative management. J Child Orthop 9(6):495–503

52. Tran P, Pritchard M, O’Donnell J (2013) Outcome of arthroscopic treatment for cam type femoroacetabular impingement in adolescents. ANZ J Surg 83(5):382–386

53. Willimon SC, Egger A, Perkins CA (2021) TRAUMATIC HIP DISLOCATIONS IN THE PEDIATRIC PATIENT: INJURY PATTERNS, NEED FOR AXIAL IMAGING, OUTCOMES, AND SELECTIVE HIP ARTHROSCOPY. J ISAKOS 6(6):449–450

54. Winge S, Winge S, Kraemer O, Dippmann C, Hölmich P (2021) Arthroscopic treatment for femoroacetabular impingement syndrome (FAIS) in adolescents-5-year follow-up. J Hip Preserv Surg 8(3):249–254

55. Wyles CC, Howe BM, Norambuena G, Yuan B, Levy BA, Trousdale RT, Sierra RJ (2017) CAM Morphology and Limited Hip Range of Motion is Associated with Early Osteoarthritic Changes in Adolescent Athletes: A Prospective Matched Cohort Study. Arthrosc J Arthrosc Relat Surg 33(10):e43–e44

56. Yen Y-M, Kim Y-J, Ellis HB, Sink EL, Millis MB, Zaltz I, Sankar WN, Clohisy JC, Nepple JJ, ANCHOR Group (2024) Risk Factors for Suboptimal Outcome of FAI Surgery in the Adolescent Patient. J Pediatr Orthop 44(3):141–146

57. Zogby AM, Bomar JD, Johnson KP, Upasani VV, Pennock AT (2021) Nonoperative Management of Femoroacetabular Impingement in Adolescents: Clinical Outcomes at a Mean of 5 Years: A Prospective Study. Am J Sports Med 49(11):2960–2967
